# Apolipoprotein CIII overexpression exacerbates diet-induced obesity due to adipose tissue higher exogenous lipid uptake and retention and lower lipolysis rates

**DOI:** 10.1186/s12986-015-0058-6

**Published:** 2015-12-23

**Authors:** Helena F. Raposo, Adriene A. Paiva, Larissa S. Kato, Helena C. F. de Oliveira

**Affiliations:** Department of Structural and Functional Biology, Institute of Biology, State University of Campinas, Campinas, SP Brazil; Instituto de Biologia, Universidade Estadual de Campinas, Rua Monteiro Lobato, 255, Campinas, SP CEP 13083-862 Brazil

**Keywords:** Adipose tissue, Hypertriglyceridemia, High-fat diet, Lipid metabolism, Transgenic animal models

## Abstract

**Background:**

Hypertriglyceridemia is a common type of dyslipidemia found in obesity. However, it is not established whether primary hyperlipidemia can predispose to obesity. Evidences have suggested that proteins primarily related to plasma lipoprotein transport, such as apolipoprotein (apo) CIII and E, may significantly affect the process of body fat accumulation. We have previously observed an increased adiposity in response to a high fat diet (HFD) in mice overexpressing apoCIII. Here, we examined the potential mechanisms involved in this exacerbated response of apoCIII mice to the HFD.

**Methods:**

We measured body energy balance, tissue capacity to store exogenous lipids, lipogenesis and lipolysis rates in non-transgenic and apoCIII overexpressing mice fed a HFD during two months.

**Results:**

Food intake, fat excretion and whole body CO_2_ production were similar in both groups. However, the adipose tissue mass (45 %) and leptin plasma levels (2-fold) were significantly greater in apoCIII mice. Lipogenesis rates were similar, while exogenous lipid retention was increased in perigonadal (2-fold) and brown adipose tissues (40 %) of apoCIII mice. In addition, adipocyte basal lipolysis (55 %) and in vivo lipolysis index (30 %) were significantly decreased in apoCIII mice. A fat tolerance test evidenced delayed plasma triglyceride clearance and greater transient availability of non-esterified fatty acids (NEFA) during the post-prandial state in the apoCIII mice plasma. Thus, apoCIII overexpression resulted in increased NEFA availability to adipose uptake and decreased adipocyte lipolysis, favoring lipid enlargement of adipose depots.

**Conclusion:**

We propose that plasma apoCIII levels represent a new risk factor for diet-induced obesity.

**Electronic supplementary material:**

The online version of this article (doi:10.1186/s12986-015-0058-6) contains supplementary material, which is available to authorized users.

## Background

Obesity is a condition in which excess body fat accumulates and may adversely affect one’s health. Body fat content is determined by interactions between genetic and environmental factors acting through the mediators of energy intake and expenditure [1, 2]. The World Health Organization estimates that in 2014 approximately 2 billion people around the world were overweight, and approximately 600 million were obese [3]. Overweight and obesity are associated with an excess of cardiovascular and non-cardiovascular deaths in the general population [4, 5].

Hypertriglyceridemia is one of the most common types of dyslipidemia found in obesity, together with low HDL levels and the presence of small and dense LDL particles [6]. Most disorders in lipoprotein metabolism in obese subjects are considered as consequences of insulin resistance [6, 7]. However, it is not established whether primary hyperlipidemia can predispose one to obesity. Experimental evidences have suggested that proteins primarily related to plasma lipoprotein transport, such as apolipoprotein (apo) C and E, may significantly influence body fat accumulation. Overall, deficiency of apoE results in diminution of diet-induced obesity [8–12]. Regarding apoC, the knockout of apoCIII seems to exacerbate diet induced obesity [13, 14], while the overexpression of apoCI protects from diet and genetic obesity [15].

ApoCIII is mainly found in triglyceride (TG)-rich lipoproteins [16, 17]. A strong positive correlation between plasma apoCIII and TG concentrations has been invariably observed in human and animal studies [18–20]. Transgenic mice overexpressing human apoCIII have marked elevated TG and non-esterified fatty acid (NEFA) levels [21]. ApoCIII overexpression in this model increases the half-life of TG-rich lipoproteins, without changing lipoprotein lipase (LPL) activity in vivo [22, 23], although this apolipoprotein may be a potent LPL inhibitor in vitro [24, 25]. In addition, growing evidence has linked apoCIII concentrations in plasma lipoproteins, including HDL, to coronary heart disease [19, 20, 26, 27].

We previously hypothesized that apoCIII overexpression would compromise fatty acid delivery to adipose tissues and would thus contribute to resistance to diet-induced obesity, similarly to what was described for overexpression of apoCI [15]. However, this was not the case, as after five months of a high fat diet, apoCIII overexpressing mice accumulated more body fat than non-transgenic littermates [28]. Therefore, the role of apoCIII on adiposity seems to be quite complex because both apoCIII overexpression or disruption results in more severe diet-induced obesity than wild type mice [14]. Therefore, the aim of the present study was to evaluate major functional and biochemical processes which could be involved in diet-induced body fat accumulation related to the excess of apoCIII.

## Methods

### Animals and treatments

Human apoCIII transgenic mice (line 3707) [29] founders were originally donated by Dr. Alan R. Tall (Columbia University, New York, NY, 1996) and crossbred with wild-type (NTg) C57BL/6 J mice. The apoCIII transgenic colony has been kept since 1996 at the animal facilities of the Division of Physiology and Biophysics at the State University of Campinas (São Paulo, Brazil). The experiments were approved by the university’s ethics committee (protocol # 1607–1). Transgenic mice were screened according to their triglyceride plasma levels (apoCIII > 300 mg/dl and controls < 100 mg/dl). All experiments were performed with female mice. ApoCIII transgenic and non-transgenic (NTg) female littermates were housed in a room at 22 °C ± 1 °C with a 12-hour light–dark cycle and had free access to water and diet *ad libitum*. From weaning, mice received standard laboratory rodent diet (CR1; Nuvital, Colombo, PR, Brazil) and a high fat diet (HFD) from 2 to 4 months of age (Additional file 1) or from 4 to 6 months of age. Mice body weight and food intake were measured weekly. At the end of the diet treatment, mice were anesthetized with ketamine and xylazine (100 and 10 mg/Kg, respectively) and killed by exsanguination through the retroorbital plexus. The perigonadal, subcutaneous (inguinal) and interscapular brown adipose tissues were excised and fresh masses were determined gravimetrically.

### Plasma biochemical analyses

The plasma levels of total cholesterol (CHOL), triglycerides (TG) (Chod-Pap; Roche Diagnostic GmbH, Mannheim, Germany), non-esterified fatty acids (NEFA) (Wako Chemicals, Neuss, Germany) and glycerol (Bioclin, Quibasa; Belo Horizonte, Brazil) were determined using enzymatic colorimetric assays according to the manufacturer's instructions. Blood glucose (GLUC) concentrations were measured using a glucose analyzer (Accu-Chek Advantage, Roche Diagnostic, Switzerland). Leptin plasma concentrations were determined by ELISA kit (Merck Millipore, Darmstadt, Germany). A fluorometric assay kit was used to determine the LPL activity (Cell Biolabs, San Diego, USA). Analyses were performed either in the fed state or after 12 h of fasting. Mice were also tested for glucose tolerance, as follows: fasted mice received an oral dose of a glucose solution (1.5 g/kg) and plasma glucose levels were determined at 0, 15, 30, 60, 90 and 120 min. Glycemia was measured with a glucometer Accu-Chek Advantage (Roche Diagnostic, Switzerland).

### In vivo CO_2_ production rates

Whole body in vivo CO_2_ production rates were measured in a temperature-monitored respirometer, as previously described [30]. Fed mice were adapted to the respirometer chamber twice a day for 5 minutes. After the adaptation period, the CO_2_ expiration of each mouse was monitored for 5 minutes once a day, between 9 AM and 11 AM, for 5 consecutive days. CO_2_ production rates were calculated as the average of the 5 measurements for each mouse and expressed as g/Kg BW/h.

### Microtomography imaging of adipose tissue

Anesthetized mice were placed in the micro-CT scanner (Bruker - Skyscan 1178). The energy parameters (49 KV; 402 μA; 20 W) were set as previously reported [31]. All images were obtained in duplicate, in 180°, in gray scale and with 84 μm of resolution. The region of interest (from cervical to tail) was determined according to the bone projections. Adipose tissue, less dense, is seen in a darker gray than more dense organs such as muscle, bone and other abdominals organs. Lung was excluded of the region of interest in each image. The 2D images were reconstructed with the NRecon software (Feldkamp algorithm). Then, images were binarized according to the established threshold to make adipose tissue show up in white.

### Adipocyte isolation

Adipocytes from fed mice were isolated using modifications of the established protocol for rat adipocytes [32]. Briefly, perigonadal and subcutaneous fat were cut into small pieces, and the fragments were digested at 37 °C with collagenase II (Sigma-Aldrich, St Louis, MO) (1 mg/ml) in Krebs-Ringer bicarbonate buffer (KRBA) containing fatty acid free albumin (3 %) and glucose (6 mM) at pH 7.4. After 45 min of incubation under continuous shaking, the fat cells were filtered through a nylon mesh and washed 3 times with KRBA to eliminate the stroma-vascular fraction and collagenase. The cells were then counted in a Neubauer`s chamber, and the viability was verified with trypan blue.

### Lipogenesis rates in isolated adipocytes

Isolated adipocytes (10^6^ cells) from fed mice were incubated in triplicate in a Krebs-Ringer phosphate buffer containing 3 % fatty acid-free BSA, 6 mM glucose, 1 mM acetate and 25 μU human insulin for 2 hours at 37 °C and saturated with a gas mixture of CO_2_ (5 %)/O_2_ (95 %) in a shaking water bath. All aliquots were incubated with 1 μCi of ^14^C-acetate (GE Healthcare-Amersham, United Kingdom). After incubation, the mixture was acidified with 0.2 ml H_2_SO_4_ (8 N) and incubated for an additional 30 min. Then, the reaction mixture was treated with 2.5 ml of Dole’s reagent (isopropanol: n-heptane: H_2_SO_4_, 4:1:0.25, v/v/v) for lipid extraction [33]. Beta radiation in the lipid extract was counted with scintillation liquid (GE Healthcare-Amersham, United Kingdom) in a Beckman - LS 6000TA Beta counter. The results are expressed as the percentage of the control group.

### Lipolysis rates in isolated adipocytes

Glycerol release rates from adipocytes to media were measured as indicators of lipolysis. The assay was performed in triplicate with Krebs-Ringer phosphate buffer containing 3 % fatty acid-free BSA and 6 mM glucose, pH 7.4. Isolated adipocytes (10^6^ cells) from fed mice were incubated with adenosine deaminase (0.2 U/ml) for 5 min in a shaking water bath at 37 °C to allow for the degradation of endogenous released adenosine, which is a potent inhibitor of lipolysis [34]. After this period, cells were incubated for 1.5 h at 37 °C in the presence or absence of isoproterenol (10^−5^ M), a beta-adrenergic receptor agonist. At the end of the incubation, the reaction was blocked on ice, and cells were carefully removed. The glycerol content of the incubation medium was measured using an enzymatic-colorimetric assay (Bioclin, Quibasa; Belo Horizonte, Brazil).

### In vivo lipolysis

Lipolysis was estimated as glycerol release in response to isoproterenol stimulation. Fed mice were injected with isoproterenol (0.3 mg/Kg, *ip*) [35]. Plasma samples were collected from the tail tip without anesthesia at basal time and 15 minutes after isoproterenol *ip* injection. The plasma glycerol concentrations were measured using an enzymatic-colorimetric assay (Bioclin, Quibasa; Belo Horizonte, Brazil). The lipolysis index is defined as the ratio between the concentrations of glycerol after and before isoproterenol stimulus.

### Fat tolerance test

Mice underwent an oral fat tolerance test as previously reported [36]. After an overnight fast, blood samples were collected before and every 2 hours after the administration of an oral dose of corn oil (10 ml/Kg of body weight), during 8 hours. Non-esterified fatty acid (Wako Chemical, Neuss, Germany) and triglycerides (Chod-Pap; Roche Diagnostic GmbH, Mannheim, Germany) were determined in plasma by enzymatic-colorimetric methods according to the manufacturers’ instructions.

### Exogenous lipid retention capacity

After 8 hours of fasting, mice received an oral dose of ^3^H-triolein (5 μCi ^3^H-TO, GE Healthcare-Amersham, United Kingdom) mixed with corn oil (180 mg/mouse). Blood samples were collected 3, 18, 20 and 24 hours to determine the plasma ^3^H activity. At 24 hours, mice were deeply anesthetized and killed by exsanguination. Liver, gastrocnemius muscle and perigonadal, subcutaneous and interscapular brown fat depots were excised and weighed. Tissue lipids were extracted using the Folch [37] method, and beta radiation was counted with scintillation liquid (GE Healthcare-Amersham, United Kingdom) in a Beckman - LS 6000TA Beta counter.

### RNA extraction and Real time – PCR

Adipose tissue RNA was extracted from 100 mg of tissue using RNeasy Lipid Tissue Mini Kit (QIAGEN, Germany), according to the manufacturers’ instructions. The integrity of the RNA was assessed using Tris-borate 1.2 % agarose gels stained with ethidium bromide. The amount and purity of the RNA were determined by optical density readings at 260 and 280 nm (Gene Quant, Amersham-Pharmacia Biotech). Genomic DNA contamination was excluded by running a polymerase chain reaction (PCR) on the RNA samples. cDNA was prepared from 2 μg of total RNA by reverse transcription using an Applied Biosystems kit (High-Capacity cDNA reverse transcription kit) according to the manufacturer's instructions. Gene expression (mRNA) was determined by real-time reverse transcription polymerase chain reaction (RT-PCR) (Step One Real-time PCR System, Applied Biosystems, Foster City, CA, USA) using a SybrGreen PCR master mix. Specific primers sequences are provided as Additional file 1: Table S1. Gene expression was quantified using the ΔΔCT method by measuring the threshold cycle normalized to β-actin and then expressed relative to the control groups.

### Statistical analysis

The results are presented as the mean ± standard error for the number of determinations (n) indicated. Student’s t-test was used for two group comparisons. Statistical significance was defined as p ≤ 0.05.

## Results

**Fig. 1:**
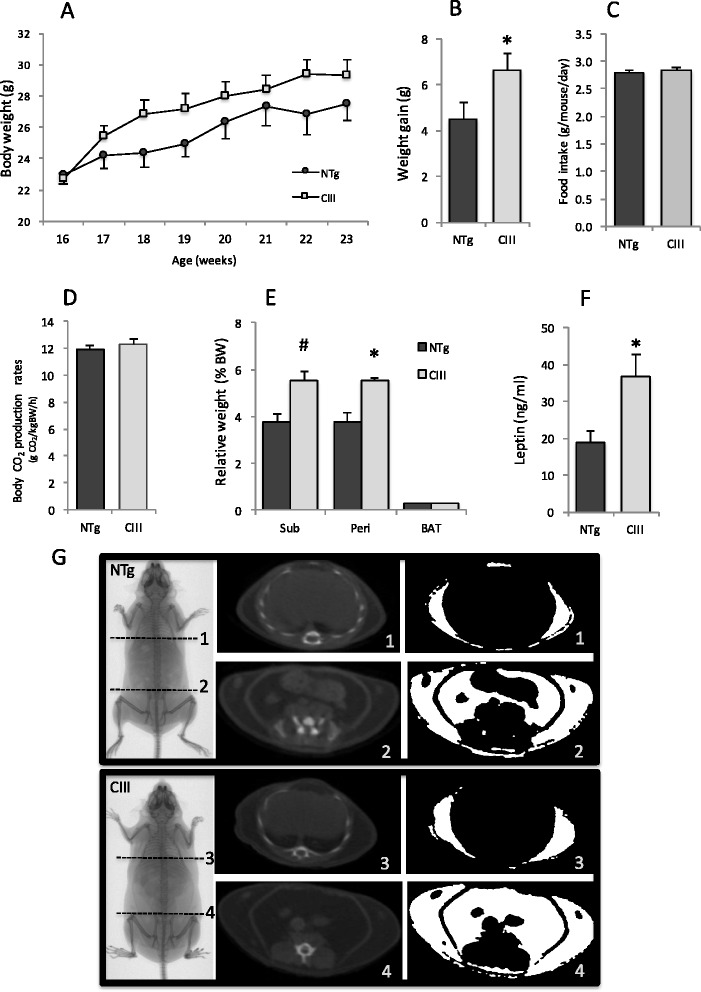
ApoCIII transgenic (Tg) mice accumulate more body fat compared with non-transgenic (NTg) mice after 8 weeks of high fat diet. Body weight (**a**), weight gain (**b**) (*n* = 6-7), daily food intake (**c**), resting metabolism (**d**) (*n* = 4), relative weight of adipose tissue depots (**e**) (*n* = 6-7) and plasma leptin levels (**f**) (*n* = 13). Mean ± SE. Student’s t test, * *P* < 0.05 and # *P* < 0.07. Representative tomography images (**g**): The less dense adipose tissue appears as darker gray than more dense organs such as muscle, bone and others (middle images). Lung was excluded of the region of interest in each image (upper right images). The images were binarized accordantly to the established threshold to make adipose tissue show up in white (right images)

In a previous study, we reported that apoCIII transgenic mice accumulated more body fat than control non-transgenic littermates after consuming a high fat diet (HFD) for 5 months since weaning [28]. In order to identify processes that contribute to the development of obesity in this animal model we investigated apoCIII transgenic and non-transgenic (NTg) littermate mice under HFD for 2 month. At first, mice were treated from 2 to 4 months of age, but apoCIII mice adipose depots were just slightly larger than NTg depots, although plasma leptin levels and adipose exogenous lipid retention capacity were significantly elevated (Additional file 1: Figure S1). Aditional groups of mice were treated with HFD from 4 to 6 months of age, and in this treatment, apoCIII mice were significantly more obese than NTg (Fig. 1). Thus, this protocol was used for further experiments to understand the mechanisms responsible for this HFD effect on apoCIII mice.

The HFD did not change the apoCIII hyperlipidemic phenotype that is already present under a low fat diet (Table 1). The apoCIII mice maintained higher plasma levels of triglycerides (~5-fold), cholesterol (~70 %) and non-esterified fatty acids (NEFA, ~100 %) when compared to NTg mice. Glycerol plasma levels did not differ in the fasting state but were higher in apoCIII than in NTg mice in the fed state. This key finding indicates that the higher glycerol levels in fed CIII mice are derived from the intravascular lipolysis of post-prandial plasma lipoproteins and do not reflect adipose tissue lipolysis (fasting). Fasted and fed glycemia levels were similar in both groups (Table 1), as well as the glucose tolerance to an oral glucose load (data not shown).

**Table 1:** Plasma levels of lipids and glucose in non-transgenic (NTg) and apoCIII transgenic mice after 8 weeks of high fat diet.

		NTg	CIII
CHOL (mg/dL)	Fed	171 ± 11.3 (6)	288 ± 36.7** (7)
TG (mg/dL)	Fast	60 ± 6.9 (4)	284 ± 51.7** (4)
	Fed	52 ± 5.6 (6)	341 ± 54.4** (7)
NEFA (nmol/L)	Fast	0.69 ± 0.15 (4)	0.97 ± 0.16 (4)
	Fed	0.7 ± 0.06 (6)	1.3 ± 0.04** (7)
Glycerol (mg/dL)	Fast	3.7 ± 0.3 (15)	4.1 ± 0.3 (13)
	Fed	3.2 ± 0.3 (15)	4.6 ± 0.4* (13)
GLUC (mg/dL)	Fast	102.4 ± 4.8 (13)	107.6 ± 3.6 (15)
	Fed	107.6 ± 3.6 (13)	114.8 ± 3.6 (15)

Triacylglycerol (TG), Cholesterol (CHOL) and Non-esterified fatty acid (NEFA). Mean ± SE (n). Student’s t test, *P ≤ 0.05 **P ≤ 0.01 for CIII *vs* NTg

After 2 months on a HFD diet, apoCIII mice showed greater body weight gain compared to NTg controls (Fig. 1a and b), although there were no significant differences in final whole body weight. Food intake (Fig. 1c) and resting metabolism (CO_2_ production, Fig. 1d) were similar between groups. However, apoCIII mice developed larger adipose tissue depots than NTg controls (Fig. 1e). The mass of the perigonadal and subcutaneous fat depots of apoCIII mice were 49 and 36 % greater than the respective NTg mice depots. Confirming increased adiposity, the leptin plasma levels in apoCIII were two-fold higher than in NTg mice (Fig. 1f). Micro-CT scanner representative images (Fig. 1g) illustrate the enhanced subcutaneous and visceral adiposity in apoCIII mice.

**Fig. 2:**
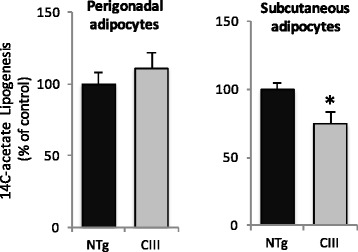
Lipogenesis rates measured in isolated adipocytes from adipose tissues in apoCIII transgenic (Tg) and non-transgenic (NTg) mice after 8 weeks of high fat diet. Adipocytes (10^6^ cells) were incubated for 2 hours at 37°C in the presence of 1 μCi ^14^C-acetate, 10 nM acetate, 25 μU insulin, and 6 mM glucose. Mean ± SE (*n* = 9) (three independent experiments)

De novo lipogenesis rates were compared in isolated adipocytes from apoCIII and NTg mice (Fig. 2). There were no significant differences in de novo lipid synthesis in adipocytes from the perigonadal adipose tissue of both groups of mice. Lipogenesis rates in the subcutaneous adipose tissue was actually decreased by 25 % in apoCIII compared to control NTg mice. Thus, this process certainly does not explain the enlargement of this adipose depot.

**Fig. 3:**
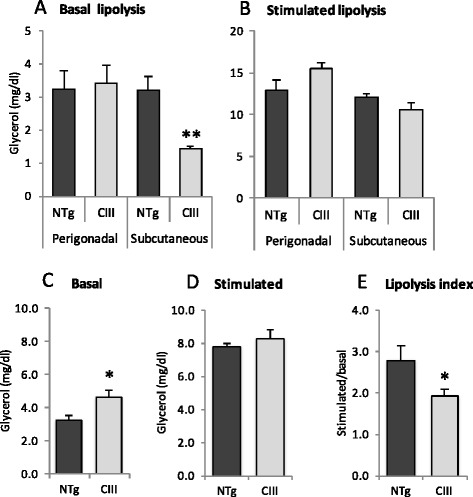
Lipolysis rates (in the basal and isoproterenol-stimulated conditions) of apoCIII transgenic (Tg) and non-transgenic (NTg) mice after 8 weeks of high fat diet measured in isolated adipocytes (**a**-**b**) (*n* = 6, two independent experiments) and in vivo (**c**-**e**) (*n* = 13-15). Adipocytes (10^6^) isolated from fed mice were incubated for 1.5 hours at 37°C in the presence of 0.2 U/ml adenosine deaminase, with or without 10^−5^ M isoproterenol. Lipolysis index in fed mice is estimated as the ratio of glycerol plasma levels in the isoproterenol-stimulated over basal conditions. Mean ± SE. Student’s t test, *P ≤ 0.05; **P ≤ 0.01

Next, lipolysis rates were measured in vitro (in isolated adipocytes) and in vivo. Basal and isoproterenol-stimulated lipolysis in isolated adipocytes from perigonadal and subcutaneous fat depots are shown in Fig. 3a. Lipolysis was determined as glycerol release to the media. Basal lipolysis in subcutaneous adipocytes was significantly reduced by 55 % in apoCIII compared to NTg adipocytes, but no differences were noted in perigonadal adipocytes. When lipolysis was maximally stimulated by isoproterenol in vitro, no significant differences were observed between adipocytes of both groups of mice. Therefore, the marked reduction in subcutaneous lipolysis may explain increased mass of this adipose depot observed in apoCIII mice (Fig. 1e). To confirm and expand these results in a more physiological context, we also estimated adipose lipolysis rates in vivo, by measuring plasma glycerol levels after isoproterenol injection, which stimulates adipose tissue hormone sensitive lipase. Because basal glycerol plasma levels in fed apoCIII mice are higher, the response to isoproterenol stimulation in vivo must be normalized by the basal levels of glycerol (Fig. 3c). Therefore, the in vivo lipolysis index is expressed as the ratio between glycerol concentrations in the stimulated and basal states. It is verified that the isoproterenol lipolytic response is significantly reduced by approximately 30 % in apoCIII transgenic mice (Fig. 3e). The possible reduction of plasma lipoprotein lipase (LPL) amount is not implicated in this result, since plasma LPL activities (measured with exogenous substrate) were similar in both groups (Table 2).

**Table 2:** Plasma lipoprotein lipase (LPL) activity in non-transgenic (NTg) and apoCIII transgenic mice.

		NTg	CIII
Chow diet	Fed	44 ± 2.4 (4)	37 ± 0.9 (5)
	Fed/post-heparin	51 ± 3.0 (4)	38 ± 1.2 (5)
HFD	Fed	40 ± 2.4 (4)	35 ± 5.4 (4)

Chow diet from weaning to 6 month of age and HFD (high fat diet) from 4 to 6 month of age. Plasma obtained in fed state before and after heparin *ip* injection (100 U/Kg BW). Mean ± SE (n). Student’s t test: no differences between groups

**Fig. 4:**
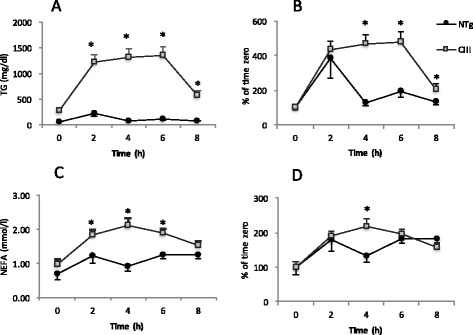
Plasma levels of triglycerides (TG, **a** and **b**) and non-esterified fatty acid (NEFA, **c** and **d**) after an oral fat load (corn oil 10 ml/Kg bw) administered to fasted apoCIII transgenic (Tg) and non-transgenic (NTg) mice that had been on a high fat diet for 8 weeks. Absolute (**a**, **c**) and incremental values (**b**, **d**). Mean ± SE (*n* = 4). Student’s t test, * *p* ≤ 0.05

**Fig. 5:**
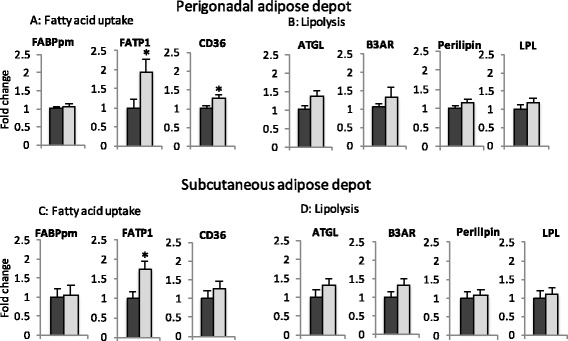
Adipose tissue mRNA expression of genes related to fatty acid uptake and lipolysis in apoCIII transgenic (Tg) and non-transgenic (NTg) mice after 8 weeks of high fat diet. Relative values normalized by β-actin mRNA. Mean ± SE (*n* = 7-9). Student’s t test, *p ≤ 0.05. ATGL (adipose triglyceride lipase), CD36/FAT (fatty acid translocase), FATP1 (fatty acid transport protein-1), UCP1 (uncoupling protein-1), B3AR (beta 3 adrenergic receptor), FABPpm (fatty acid binding protein- plasma membrane)

In order to understand whether apoCIII would alter the response to fat intake, a fat tolerance test was performed. Increases in TG and NEFA plasma levels after the fat load are shown as absolute and relative plasma values (Fig. 4a-d). The similarities in curves slopes up to 2 hours indicate that fat absorption rates are similar between both groups, regardless the basal plasma TG levels. However, 4 hours after the oral oil dose, plasma TG reduces rapidly in the NTg group, but not in the apoCIII group, which characterizes the delay in TG-rich lipoproteins clearance caused by the apoCIII overexpression. It is important to mention that under HFD there is no alteration of TG liver production rates in control and apoCIII mice [38]. After 8 hours, TG plasma levels return to their respective basal levels in both groups. The NEFA curves (Fig. 4c) indicate a greater availability of these substrates during the post-prandial state, from 2 to 6 hours after the oil dose, in the apoCIII mice plasma compartment. Interestingly, we found higher expression of FATP1 and CD36 mRNA, both associated with fatty acid uptake in the adipose depots of apoCIII mice (Fig. 5). Regarding genes related to adipose tissue lipolysis, there were no differences in adipose tissue ATGL (adipose triglyceride lipase), beta 3-adrenergic receptors, perilipin and LPL mRNA levels between groups (Fig. 5).

**Fig. 6:**
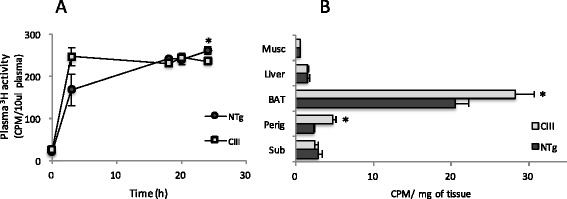
In vivo exogenous lipid retention in adipose, liver and muscle tissues of apoCIII transgenic (Tg) and non-transgenic (NTg) mice after 8 weeks of high fat diet. Plasma (**a**) and tissue (**b**) ^3^H-lipid radioactivity were followed after an oral dose of ^3^H-triolein (5 μCi ^3^H-triolein, 180 mg/mouse). Mean ± SE (*n* = 7). Student’s t test, * *p* ≤ 0.05

To evaluate adipose tissue capacity to take up and retain lipids, mice were challenged with an oral dose of corn oil containing the tracer ^3^H-triolein. The time course of plasma radioactivity and tissue retention after 24 hours are shown in Fig. 6. Like already observed in the fat tolerance test, there is a delay in the exogenous TG plasma clearance in apoCIII mice, observed as an increase in their plasma levels of ^3^H-TG after 3 hours, which was normalized at 18 and 20 hours and decreased at 24 hours (Fig. 6a). At this time point (24 hours), tissues were collected and the lipid extraction data demonstrates a higher retention of the TG tracer in the perigonadal and brown adipose tissues of apoCIII mice (Fig. 6b).

## Discussion

In this work we examined the potential principal mechanisms involved in the increased adiposity in response to five months of high fat diet in mice overexpressing apoCIII [28]. We confirmed the previous observed apoCIII fatter phenotype even in a shorter (2 months) period of high fat diet. The food ingestion and body energy expenditure cannot explain this enlarged body fat mass, since these processes were similar in apoCIII and NTg mice. However, the weight gain, mass of adipose tissues and leptin plasma levels were significantly greater in apoCIII mice. The results show that this exacerbated HFD response in apoCIII mice can be explained by an increased exogenous (diet) lipid retention capacity in adipose tissue and reduced adipose lipolysis rates in apoCIII mice. Lipid deposition in non-adipose tissues seems not to occur in these mice, since lipid retention in liver and skeletal muscle were similar to control NTg mice, at least in this relatively short time of HFD. Therefore, data presented here demonstrate that apoCIII overexpression favors an increased lipid retention (balance between uptake and lipolysis) in adipose depots. We propose that this is an adaptation to the marked greater availability of post-prandial NEFA derived from long lasting circulating TG rich lipoproteins that are taken up and stored by the adipose tissues.

Previous studies (reviewed in ref [39]) showed that modulation of LPL activity through its activators [9, 10] or inhibitors [14, 15] may have effects on triglyceride storage. For instance, LPL deficiency in adipose tissue reduced adiposity in obese mice [40], while its overexpression in muscle resulted in increased TG content in this tissue [41, 42]. In addition, apoCI transgenic mice present lower LPL activity and are protected against obesity [15]. On the contrary, we show here (Table 2) and elsewhere [43] that apoCIII overexpression does not change plasma LPL activities and still results in diet induced obesity. Nowadays, it has become clear that apoCIII inhibition of LPL requires a very high CIII/CII ratio, which is not found in vivo [26]. Indeed, Aalto-Settala et al. [22] showed that VLDL from apoCIII and NTg mice are equally hydrolyzed in vitro by purified LPL. Accordingly, the higher glycerol and NEFA post-prandial plasma levels in apoCIII mice (Table 1, Fig. 4) indicate no inhibition of plasma TG rich lipoprotein lipolysis. If LPL were inhibited in apoCIII mice, the availability of NEFA to adipose tissues would be severely limited and increased adiposity would not occur as shown here (Fig. 1) and previously [28]. Therefore, we rule out a role of LPL to explain increased obesity in apoCIII mice. Instead, the higher substrate availability (Fig. 4), lower adipose lipolysis (Fig. 3) and higher adipose tissue uptake/retention capacity (Fig. 6) are the key factors implicated in the diet induced obesity in apoCIII mice.

The detailed molecular pathways underlying the induction of fat accumulation by overexpression of apoCIII were not addressed in this study. It is well known that fatty acids can modulate intracellular signaling pathways by changing cell membrane fluidity, the composition of lipid rafts and second messengers production. In addition, they can act on receptors either in the cell membrane or in the nucleus, such as the toll-like receptors (TLRs) and the peroxisome-proliferator-activated receptors (PPARs), respectively. We propose that apoCIII induced high NEFA availability could modulate specific protein activities or gene expression related to lipid uptake, accumulation and lipolysis in the adipose tissue of apoCIII mice. In fact, the adipose tissue FATP1 and CD36 expression were higher in apoCIII mice, suggesting that fatty acid uptake may be increased in these mice. Other effects on lipid accumulation and intracellular lipolysis observed here might be the result of direct modulation of key proteins activity, without modifying necessarily the gene expression. For instance, those triggered by isoproterenol stimulation of intracellular lipolysis, which are mediated by the B3R-AMPc-PKA-HSL pathway [44]. In addition, it has been suggested that apoCIII per se, independently of TG/NEFA levels, stimulates inflammatory processes in the vasculature [20, 45]. Thus, one could speculate that apoCIII induced inflammation may have hampered tissue lipolysis and facilitated lipid accumulation in adipocytes.

## Conclusion

Together the results indicate that apoCIII overexpression exacerbates diet-induced obesity by promoting increased availability of NEFA from post-prandial TG-rich lipoproteins combined with greater adipose capacity for lipid uptake and retention and reduced adipose lipolysis. Therefore, this study revealed a new risk factor for susceptibility to obesity that can be attributed to high levels of apoCIII on top of those already reported for atherosclerosis and vasculature inflammation.

## Additional file

Additional file 1:
**Supplemental data.** (DOCX 94 kb)

## Abbreviations

Apo: Apolipoprotein
ATGL: Adipose triglyceride lipase
B3AR: Beta-3 adrenergic receptor
CHOL: Cholesterol
CD36: (Cluster of Differentiation 36), FAT (fatty acid translocase)
FABPpm: Plasma membrane fatty acid binding protein
FATP: Fatty acid transport protein
HFD: High fat diet
IP: Intraperitoneal
KRBA: Krebs-Ringer bicarbonate buffer
LPL: Lipoprotein lipase
NTg: Non-transgenic
PPARs: Peroxisome-proliferator-activated receptors
TG: Triglycerides
TLR: Toll-like receptors
TO: Triolein
