# Supplementary material for: Apolipoprotein CIII overexpression exacerbates diet-induced obesity due to adipose tissue higher exogenous lipid uptake and retention and lower lipolysis rates
Source: Nutr Metab (Lond). 2015 Dec 23;12:61. doi: 10.1186/s12986-015-0058-6 (PMC4690294; doi:10.1186/s12986-015-0058-6)
Supplement: Additional file 1: — Supplemental data. (DOCX 94 kb) [file 12986_2015_58_MOESM1_ESM.docx]

**SUPLEMENTAL DATA**

**Table S1.** Real-time PCR primers list

| Gene | Primers | |
| --- | --- | --- |
| β-actin | forward | 5` GGACTCATCGTACTCCTGCTT 3` |
|  | reverse | 5` GAGATTACTGCTCTGGCTCCT 3` |
| ATGL | forward | 5` TGTGGCCTCATTCCTCCTAC C 3` |
|  | reverse | 5` TCGTGGATGTTGGTGGAGCT 3` |
| CD36 | forward | 5` GGAACTGTGGGCTCATTGC 3` |
|  | reverse | 5` CATGAGAATGCCTCCAAACAC 3` |
| FATP1 | forward | 5` GCAGAAGACGCAGGAAGA 3` |
|  | reverse | 5` GGACGTGGCTGTGTATGG 3` |
| Perilipin | forward | 5` GTACACTATGTGCCGCTTCC 3` |
|  | reverse | 5` CTTTGCGCTCCGCCTCT 3` |
| UCP1 | forward | 5` GATGGTGAACCCGACAACTT 3` |
|  | reverse | 5` CTGAAACTCCGGCTGAGAAG 3` |
| B3AR | forward | 5` GGCAACCTGCTGGTAATCAT 3` |
|  | reverse | 5` TCCACTGACGTCCACAGTTC 3` |
| FABPpm | forward | 5` ACTTGGTGACCTGGTGAATG 3` |
|  | reverse | 5` CAGATCGGCATGTTCTGTTT 3` |

ATGL (Adipose triglyceride lipase), CD36/FAT (fatty acid translocase), FATP1 (fatty acid transport Protein-1), UCP1 (uncoupling protein-1), B3AR (beta 3 adrenergic receptor), FABPpm (fatty acid binding protein- plasma membrane).

**Table S2.** Fat intake and excretion (g/day/mouse) in non-transgenic (NTg) and apoCIII transgenic mice fed a high fat diet (HFD) from two to four months of age.

|  | **NTg** | **CIII** |
| --- | --- | --- |
| Fat intake | 0.8 ± 0.04 | 0.8 ± 0.01 |
| Fecal mass | 0.42 ± 0.03 | 0.40 ± 0.02 |
| Fat excretion (% dry feces) | 4.0 ± 0.36 | 4.4 ± 0.03 |

Feces collected during 3 days were dried, and their fat content was extracted by the Folch (1957) method to determine the amount of fat excretion. Mean ± SE (n= 6-8).

**Figure S1**: ApoCIII transgenic (Tg) mice fed a high fat diet (HFD) from two to four months of age show increased exogenous lipid retention and plasma leptin levels compared with non-transgenic (NTg) littermates. Weight gain (A), relative weight of adipose tissue visible depots and liver (B) (n=21), leptin plasma levels (C), daily food intake (D), body CO_2_ production rates (E) (n=7-6), tissue lipid retention 24 hours after an oral dose of ^3^H-triolein (5 μCi ^3^H-triolein, 180 mg/mouse) (F) (n=8-7). Mean ± SE. Student’s t test: * *P*<0.05; # *P*=0.056.
